# Hydrogel Microwell Arrays Allow the Assessment of Protease-Associated Enhancement of Cancer Cell Aggregation and Survival

**DOI:** 10.3390/microarrays2030208

**Published:** 2013-08-22

**Authors:** Daniela Loessner, Stefan Kobel, Judith A. Clements, Matthias P. Lutolf, Dietmar W. Hutmacher

**Affiliations:** 1 Faculty of Health, Institute of Health and Biomedical Innovation (IHBI), Queensland University of Technology (QUT), 60 Musk Avenue, Kelvin Grove 4059, Brisbane, Australia; E-Mails: daniela.lossner@qut.edu.au (D.L.); j.clements@qut.edu.au (J.A.C.); 2 Laboratory of Stem Cell Bioengineering, Institute of Bioengineering, École Polytechnique Fédérale de Lausanne (EPFL), Building AI 3138, Station 15, CH-1015 Lausanne, Switzerland; E-Mails: stefan.kobel@roche.com (S.K.); matthias.lutolf@epfl.ch (M.P.L.); 3 Faculty of Science and Engineering, IHBI, QUT, 60 Musk Avenue, Kelvin Grove 4059, Brisbane, Australia

**Keywords:** microwell arrays, cell aggregates, bioengineered microenvironments, ovarian cancer, kallikreins, integrins, paclitaxel

## Abstract

Current routine cell culture techniques are only poorly suited to capture the physiological complexity of tumor microenvironments, wherein tumor cell function is affected by intricate three-dimensional (3D), integrin-dependent cell-cell and cell-extracellular matrix (ECM) interactions. 3D cell cultures allow the investigation of cancer-associated proteases like kallikreins as they degrade ECM proteins and alter integrin signaling, promoting malignant cell behaviors. Here, we employed a hydrogel microwell array platform to probe using a high-throughput mode how ovarian cancer cell aggregates of defined size form and survive in response to the expression of kallikreins and treatment with paclitaxel, by performing microscopic, quantitative image, gene and protein analyses dependent on the varying microwell and aggregate sizes. Paclitaxel treatment increased aggregate formation and survival of kallikrein-expressing cancer cells and levels of integrins and integrin-related factors. Cancer cell aggregate formation was improved with increasing aggregate size, thereby reducing cell death and enhancing integrin expression upon paclitaxel treatment. Therefore, hydrogel microwell arrays are a powerful tool to screen the viability of cancer cell aggregates upon modulation of protease expression, integrin engagement and anti-cancer treatment providing a micro-scaled yet high-throughput technique to assess malignant progression and drug-resistance.

## 1. Introduction

Three-dimensional (3D) *in vitro* culture approaches mimic more closely the physiological cell-cell and cell-extracellular matrix (ECM) interactions seen *in vivo* [1,2,3,4,5,6,7]. We have demonstrated that biomimetic hydrogels can be used as 3D cell culture platform to investigate the interplay of ovarian cancer cells with the ECM [8]. Within these synthetic microenvironments ovarian cancer cells form multi-cellular spheroids, an integral step leading to metastatic outgrowth and ultimately malignant progression *in vivo*. That is, after shedding from the primary tumor, these cells aggregate in order to survive within the abdominal cavity and to escape anti-cancer therapies [9,10]. Little is known about the events promoting ovarian cancer progression and how therapy-resistance occurs [11,12].

Cancer-associated proteases play a crucial role during disease progression [13]. Kallikrein-related (KLK) peptidases are known to contribute to metastatic outgrowth by modification of the tumor microenvironment via degradation of (non-)ECM proteins leading to altered cell-cell and cell-ECM interactions, cell proliferation and survival [14,15,16,17,18,19,20]. Elevated expression of KLK4, KLK5, KLK6, and KLK7 are linked to multi-cellular aggregation of ovarian cancer cells and non-responsiveness of patients to paclitaxel [21,22,23,24,25,26,27]. We have reported that combined expression of KLK4, KLK5, KLK6, and KLK7 in OV-MZ-6 ovarian cancer cells regulates integrin expression, cell adhesion, and promotes a malignant phenotype [28,29]. Of interest to this study is that integrins and integrin-related factors regulate tumor-ECM interactions leading to multi-cellular aggregation and drug-resistance [30,31,32]. Different integrins, in particular β1 integrins, are up-regulated in the advanced stages of the disease and mediate aggregation of ovarian cancer cells and therapy-resistance in patients [33,34,35]. Hence, a concomitant KLK4, KLK5, KLK6, and KLK7 expression might facilitate disease progression and lack of therapy response given that KLKs degrade ECM proteins, and therefore, influence the ECM-integrin binding dynamics.

Bioengineered microenvironments have proven to be effective in screening the responsiveness of ovarian cancer cells to paclitaxel, thereby revealing increased survival rates after paclitaxel administration in 3D compared to flat cell cultures [8]. However, 3D systems which allow cell growth upon encapsulation of single cells within a hydrogel material lead often to the formation of different sized spheroids [8]. Hence, the purpose of this study was to allow OV-MZ-6 ovarian cancer cell aggregation of a defined size layered on top of polyethylene glycol-based hydrogel microwell arrays and to assess the efficacy of paclitaxel treatment dependent on aggregate size. Furthermore, we sought to determine the contribution of combined KLK4, KLK5, KLK6, and KLK7 expression and integrins to in OV-MZ-6 cell aggregation and survival upon paclitaxel treatment employing hydrogel microwell arrays as high-throughput microarray platforms [36,37] by performing time-lapse and confocal laser scanning microscopy as well as quantitative image, gene and protein analyses dependent on varying microwell and aggregate size.

## 2. Experimental Section

***Fabrication of Hydrogel Microwell Arrays.*** The fabrication of hydrogel microwell arrays was a multistep soft lithography process as reported previously [36]. Briefly, a topographically structured silicon wafer was fabricated, and then polydimethylsiloxane (PDMS; Dow Corning Corporation, Midland, MI, USA) was cast onto this structure, and finally, hydrogel films were patterned in a stamping step using the PDMS template. A 4-inch silicon wafer was designed using the layout editor of CleWin (PhoeniX, Enschede, The Netherlands). A pattern was selected consisting of eight squares; each square matched the dimensions of a standard 96-well plate, comprising 33 × 33 = 1,000 microwells, with a diameter of 100 µm and a depth of 50 µm per microwell. Additionally, new silicon wavers were designed to produce microwells of varying sizes of 50 × 50, 100 × 100, 150 × 150, 200 × 200 µm. Microwell arrays were formed from polyethylene glycol (PEG) hydrogel precursors by cross-linking two multi-arm PEG macromers (NOF Corporation, Tokyo, Japan), end-functionalized with either thiol (SH) or vinylsulfone (VS) groups [36]. The 8arm-PEG-VS was dissolved in 0.3 M triethanolamine (Sigma-Aldrich, Buchs, Switzerland), and the 4arm-PEG-SH was dissolved in bi-distilled water to obtain 100 µm thin hydrogel films (5% (w/v)) coated onto 8-well chamber µ-slides (ibidi GmbH, Munich, Germany) for a microwell size of 50 × 100 µm or onto 48-well tissue culture plates (Thermo Fisher Scientific Inc., Lausanne, Switzerland) for a microwell size of 50–200 × 50–200 µm. Optional, hydrogel microwell arrays were coated with laminin (0.1 mg/mL; BD Biosciences, Allschwil, Switzerland) or type I collagen (0.1 mg/mL; Sigma-Aldrich), both modified with an N-hydroxylsuccinimide (NHS)-PEG-maleimide linker (JenKem Technology, Allen, TX, USA) as described previously [38].

**Figure 1:**
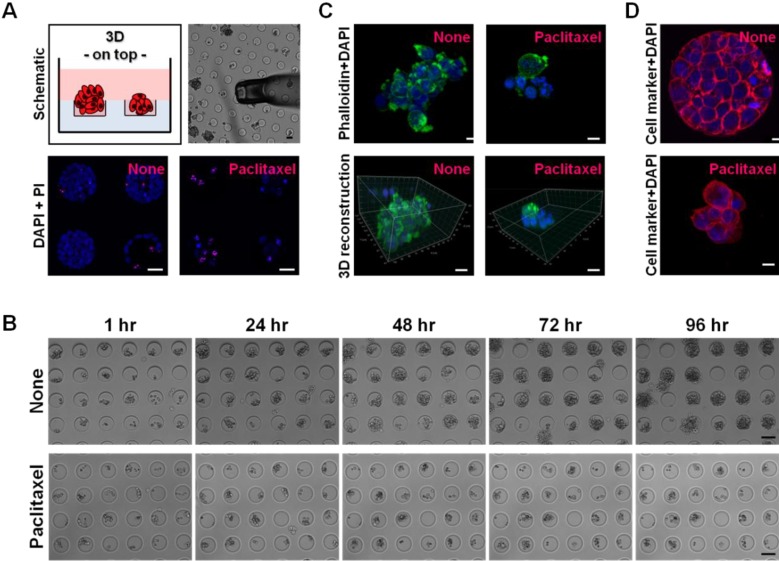
Schematic illustration and image analyses of hydrogel microwell arrays. (**A**) Cancer cell aggregates within microwells and their collection for subsequent expression analyses using a microinjector depicted by bright field microscopy (top panel). Confocal microscopy of four cell aggregates grown over 96 h within microwells (100 × 50 µm) ± paclitaxel treatment (100 nM); nuclei stained blue with DAPI; dead cells stained red with PI (bottom panel). Scale bars, 30 µm. (**B**) Time-lapse microscopy of cell aggregation ± paclitaxel treatment was performed over 96 h within microwells. Scale bars, 100 µm. (**C**) Confocal microscopy of cell aggregates grown over 96 h within microwells ± paclitaxel treatment and 3D reconstructions using Imaris; F-actin filaments stained green with Alexa488-conjugated phalloidin; nuclei stained blue with DAPI. Scale bars, 10 µm. (**D**) Confocal microscopy of the morphological marker N-cadherin in cell aggregates ± paclitaxel treatment; N-cadherin stained red using a respective primary and secondary Alexa555-conjugated IgG; nuclei stained blue with DAPI. Scale bars, 10 µm.

***Cell Aggregate Cultures.*** The human epithelial ovarian carcinoma cell line OV-MZ-6 was established from malignant tumor fluid (ascites) [39], and stable transfectants, with human KLK4, KLK5, KLK6, and KLK7 full-length cDNA (“OV-KLK”) derived from ovarian cancer tissue and an empty vector plasmid (“OV-Vector”), provided by Viktor Magdolen (Technical University of Munich, Munich, Germany), were cultured as reported previously [29]. At a confluency of 60–80%, cells were harvested with EDTA (0.48 mmol/L; Invitrogen, Lucerne, Switzerland). For cell aggregate cultures, cells (5 × 10^4^ cells/mL) were seeded on top of each square, centrifuged at 800 rpm for 5 min and grown over 120 h in 0.25 mL media (Figure 1(A)). Cell density was adapted accordingly to microwells of varying sizes (100 × 50 µm: 5 × 10^4^ cells/mL, 50 × 50 µm: 5 × 10^4^ cells/mL, 100 × 100 µm: 10 × 10^4^ cells/mL, 150 × 150 µm: 15 × 10^4^ cells/mL, 200 × 200 µm: 20 × 10^4^ cells/mL). For exposure to paclitaxel, a microtubule-stabilizing agent that mediates cell cycle arrest and apoptosis [40], cell aggregates were treated with media containing paclitaxel (0, 1, 10, 100 nM; Invitrogen). Integrin inhibition was achieved using media supplemented with a functional blocking β1 integrin antibody (10 µg/mL; Chemicon/Millipore AG, Zug, Switzerland).

***Time-Lapse Microscopy.*** Time-lapse microscopy of hydrogel microwell arrays of varying size was performed to live image cell aggregation and survival as reported previously [36]. Samples were imaged 24 h after seeding using an inverted microscope (Zeiss Axio Observer.Z1 and Zeiss Axiovert) equipped with a motorized scanning stage under sterile humidified atmosphere at 37 °C/5% (v/v) CO_2_ over 96 h, with images taken every 6 h using a 10× air objective (Figure 1(B); Supplementary file). The resulting phase contrast images were then automatically compiled into a stack using Metamorph (Molecular Devices, Sunnyvale, CA, USA). To identify dead cells, propidium iodide (PI; 1:1,000; Invitrogen) was added to the media and fluorescently imaged at the end of each experiment. Cell aggregates were grown within different sized microwells and visualized at up to 20 different positions per condition.

***Calculation of Cell Aggregate Area and Number.*** For cell aggregate number and area calculations, the integrated morphometry analysis tool in Metamorph or ImageJ ([41]) was applied to trace the aggregation number and area using either stacked bright field images or fluorescently labelled aggregates. Maximal projections using separate channels of bright field or fluorescent images were arithmetic processed, set to auto-threshold and gray levels binarized. An integrated morphometry analysis was performed to graphically identify the aggregate area. The aggregate number per microwell indicates the ratio of the number of aggregates per microwell to the number of microwells (seeding efficacy of 75–82%) counted per condition. Averages and standard errors were calculated using Excel (Microsoft, Redmond, WA, USA). For each experiment, 20 different positions per condition containing 60–960 aggregates were analyzed. For calculation of cell aggregation after paclitaxel treatment, only intact, non-lyzed cells (without the appearance of apoptotic bodies) were taken into account. Data are expressed as “relative aggregation (%)”, describing the ratio of the number of aggregates to the number of microwells analyzed per condition, and “relative cell death (%)”, referring to the ratio of the number of aggregates containing death cells (as indicated by PI staining) to the number of viable aggregates (no PI staining).

***Confocal Laser Scanning Microscopy* (*CLSM*)*.*** Cell aggregate cultures were processed as described earlier [8]. Briefly, after 4% (w/v) paraformaldehyde (PFA)/PBS containing 0.1% (v/v) triton-X100 for 30 min, F-actin filaments were stained with Alexa488-conjugated phalloidin (0.1 U/mL; Invitrogen) or rhodamine415-conjugated phalloidin (0.3 U/mL; Invitrogen) and nuclei with a far-red DNA stain (DRAQ5; 5 µM; Alexis Biochemicals/Enzo Life Sciences, Lausen, Switzerland) or 4′6-diamidino-2-phenylindole (DAPI; 2.5 µg/mL; Invitrogen) in 1% (w/v) bovine serum albumin (BSA; Sigma-Aldrich)/PBS for 1 h each at room temperature (Figure 1(C)). For cell marker staining, primary (N-cadherin (1:100; R&D Systems, Minneapolis, MN, USA)) and secondary (Alexa555-conjugated sheep IgG (1:500; Invitrogen)) antibodies 1% (w/v) BSA/PBS were incubated for 1 h each at room temperature (Figure 1(D)). Immunofluorescence was visualized and imaged using a confocal microscope (Leica TCS SP2) with a 20/40× immersion oil objective at three to five different positions per sample covering one to four aggregates. Z-stacks were acquired with constant thickness of 2 µm reconstructing a cross-section profile of 100–150 equidistant XY-scans using the Leica Microsystems LAS AF software to generate maximal projections. 3D reconstructions were built using Imaris ([42]).

***Real-Time Reverse Transcription Quantitative PCR* (*RT-qPCR*)*.*** Equal amounts (1 µg) of total RNA from cell aggregate cultures (extracted using an RNeasy micro kit; Qiagen, Magden, Switzerland) were used for cDNA synthesis. RT-qPCR was performed in triplicate with SYBR^®^ Green chemistry (AB Applied Biosystems/Life Technologies, Compark Circuit, VIC, Australia) on an ABI7300 thermal cycler (AB Applied Biosystems). Reaction setup, using an annealing temperature of 60 °C and 40 cycles, and normalization applying the standard curve method (R^2^ = 0.96–0.99) were conducted as reported previously [8]. Gene specific primers: *ITGA5*—forward 5′-catttccgagtctgggccaa-3′, reverse 5′-tggaggcttgagctgagctt-3′; *ITGB1*—forward 5′-aggtggtttcgatgccatcat-3′, reverse 5′-aagtgaaacccggcatctgtg-3′; *PTK2/FAK*—forward 5'-GCGCTGGCTGGAAAAAGAGGAA-3', reverse 5'-TCGGTGGGTGCTGGCTGGTAGG-3′; *18S*—forward 5′-gatccattggagggcaagtct-3′, reverse 5′-ccaagatccaactacgagcttttt-3′.

***Western Blotting.*** Lysates from cell aggregate cultures were collected in lysis buffer (according to RNeasy micro kit, Qiagen) as described earlier [8]. Protein concentrations were determined using protein detection reagents (bicinchoninic acid; Sigma-Aldrich) and 40 μg electrophoresed on 10% SDS-PAGE, transferred onto nitrocellulose membranes and treated with Odyssey^®^ blocking buffer (LI-COR Biosciences, Lincoln, NE, USA). Membranes were incubated with primary (α5 integrin (1:1,000; Chemicon); β1 integrin (1:1,000; Chemicon); caspase8 (1:2,000; BD Biosciences); MT1-MMP (1:500; Chemicon); GAPDH (1:10,000; Abcam, Waterloo, NSW, Australia)) and secondary (IRDye 680/800-conjugated rabbit/mouse IgG (1:5,000; LI-COR Biosciences)) antibodies overnight at 4 °C and 1 h at room temperature, respectively. Images were obtained using the Odyssey^®^ system (LI-COR Biosciences) and densitometrically evaluated.

***Statistics.*** Statistical analyses were carried out using ANOVA and Student’s t-test with “R”; results with p-values less than 0.05 were considered to be statistically significant (^*^/**^#^**—*P* < 0.05; ^**^/**^##^**—*P* < 0.01; ^***^/**^##^**—*P* < 0.001).

## 3. Results and Discussion

### 3.1. Hydrogel Microwell Arrays Allow the Aggregation of Ovarian Cancer Cells

We sought to apply high-throughput assays—to our knowledge for the first time—to allow defined aggregation of ovarian cancer cells and monitored this cellular process by confocal laser scanning microscopy (Figure 1(A,C,D)) and live cell microscopy over 96 h (Figure 1(B)) to establish their suitability as a drug screening tool using the clinically applied anti-cancer drug paclitaxel.

Cancer cells cultured as single cell suspension (1 × 10^4^ cells/mL) did not form aggregates on top of 3D cultures within microwells, and underwent only one cell division within the first 36 h after seeding (data not shown). Microwells coated with laminin or type I collagen did not increase the cell survival rates of single cell suspensions over 96 h (data not shown). As ovarian cancer cells aggregate in the tumor fluid (ascites) accumulated within the abdominal cavity of patients with advanced disease [10], we increased the number from single cancer cells per microwell (100 × 50 µm) to 5 × 10^4^ cells/mL. Time-lapse and confocal laser scanning microscopy revealed compact aggregate formation after 96 h of 3D culture with negligible cell death as indicated by minor propidium iodide (PI) staining. Upon paclitaxel treatment (100 nM), cell aggregation was dramatically reduced and scattered and cell death increased as indicated by a positive PI staining (Figure 1(A,B)). 3D reconstructions and immunostaining of the morphological marker N-cadherin confirmed compact aggregation without treatment and scattered aggregation upon paclitaxel treatment with the appearance of apoptotic nuclei (Figure 1(C,D)). These results suggest that hydrogel microwell arrays allow cancer cell aggregation.

The multi-cellular aggregate population in human ovarian tumor fluid (ascites) is thought to be a critical source for intra-abdominal metastases, and thereby, represents a key target for anti-metastatic interventions. Currently, most chemotherapies are ineffective in preventing aggregate dissemination, and the biological mechanisms leading to their formation remain poorly understood [9,10,43]. To improve our understanding of ovarian cancer biology, controlled *in vitro* models are needed to accurately mimic the *in vivo* conditions seen in patients [44]. Ill-advisedly, the terms aggregate and spheroid are inconsistently used throughout the literature, and yet, this definition is critical to the rationale of experimental 3D model approaches. The term aggregate is primarily but not always used to describe and eventually to discriminate loose packages of cells from compact spherical cultures. Aggregates with a size smaller than 150 µm may exhibit cell-cell and cell-matrix interactions. Spheroids comprise a defined cell mass of uniform geometry and physiological gradients at diameters ranging from 200–500 µm that can be manipulated and suited for large scale approaches in preclinical drug testing routines [45]. Both aggregate and spheroid cultures are well suited for developing high-throughput screening technologies [38,45], and their gene expression profiles are more truly indicative of clinical expression profiles than those detected in flat cell cultures [38,45,46]. Flat cell cultures fail to reproduce crucial aspects of carcinogenesis, such as 3D growth and architecture, cell-cell associations and cellular heterogeneity of *in vivo* samples. In this study, we have provided proof that bioengineered arrays represent a high-throughput platform reflecting 3D growth conditions of ovarian cancer cells and validated their responses by applying a clinically used therapeutic concept *in vitro*. Ovarian cancer cells grew as floatage-independent as multi-cellular aggregates. Immunostaining of structural components indicated cell-cell interactions within aggregates promoting cell survival. This microarray platform has also been used to re-create biophysical and biochemical microenvironmental cues that control stem cell fate [38], further underlining the suitability of this *in vitro* assay as a powerful 3D culture model.

### 3.2. KLK-Expressing Cells Increase Aggregation and Viability upon Paclitaxel Treatment

**Figure 2:**
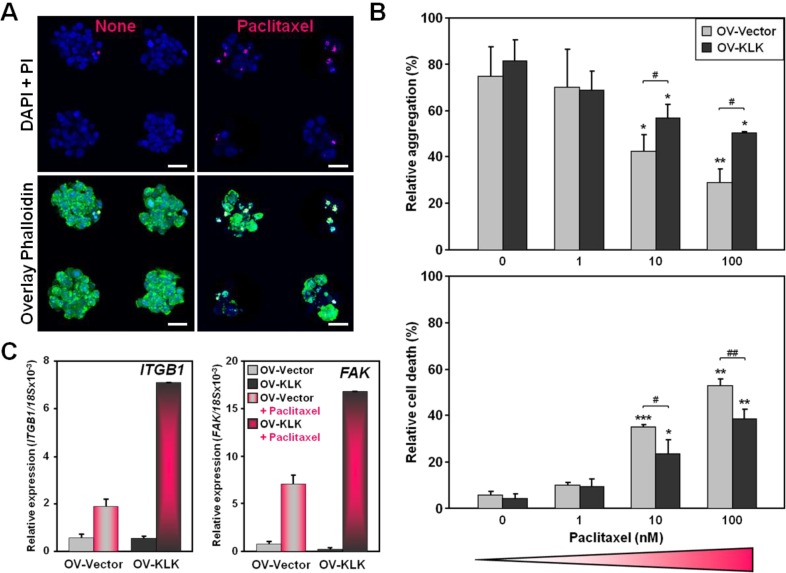
Cell aggregation, survival and gene expression upon KLK expression and paclitaxel treatment. (**A**) Confocal microscopy of four cell aggregates grown over 96 h within microwells (100 × 50 µm) ± paclitaxel treatment (100 nM); nuclei stained blue with DAPI; dead cells stained red with PI (top panel); overlaid with Alexa488-conjugated phalloidin to stain F-actin filaments, with dead cells appearing yellow (bottom panel). Scale bars, 30 µm. (**B**) OV-Vector and OV-KLK cell aggregates were grown over 96 h within microwells and treated with increasing concentrations of paclitaxel (0, 1, 10, 100 nM). Both OV-Vector/OV-KLK cells grew less aggregates at higher paclitaxel concentrations (10, 100 nM) compared to a lower paclitaxel dose (1 nM) and non-treated controls (top panel). Cell death was increased at higher paclitaxel concentrations (10, 100 nM) compared to a lower paclitaxel dose (1 nM) and non-treated controls (bottom panel; n = 3; SEM; * *P* < 0.05; ** *P* < 0.01; *** *P* < 0.001). OV-KLK cells formed more aggregates and showed less cell death at higher paclitaxel concentrations (10, 100 nM) than OV-Vector cells (n = 3; SEM; **^#^**: *P* < 0.05; **^##^**: *P* < 0.01). (**C**) Administration of paclitaxel (100 nM) was reflected in increased *ITGB1* and *FAK* levels in both OV-Vector/OV-KLK cell aggregates, with an upregulation upon KLK expression.

As cancer-associated proteases like kallikrein-related (KLK) peptidases have been attributed to chemoresistance—in particular to taxane-based drugs—in ovarian cancer [23,26,27], we further sought to investigate the effect of paclitaxel using gradually increasing doses (0–100 nM) on cell aggregation. Confocal micrographs represented the aggregate morphology with and without paclitaxel treatment (100 nM): large and compact aggregates were formed in non-treated conditions, whereas paclitaxel exposure caused smaller and scattered aggregates and the presence of apoptotic bodies. Paclitaxel treatment was correlated with a positive PI staining, indicating an increased cell death (Figure 2(A)). Both OV-Vector/OV-KLK cells formed significantly fewer aggregates at higher paclitaxel concentrations (10 nM: OV-Vector 47 ± 8%/OV-KLK 54 ± 11%; 100 nM: OV-Vector 44 ± 8%/OV-KLK 60 ± 6%) compared to a lower dose (1 nM; OV-Vector 56 ± 14%/OV-KLK 56 ± 7%) and non-treated controls (0 nM: OV-Vector 57 ± 10%/OV-KLK 61 ± 8%). Strikingly, OV-KLK cells grew significantly more aggregates at higher paclitaxel concentrations (10, 100 nM) than OV-Vector cells (Figure 2(B), top panel). Cell death in both OV-Vector/OV-KLK cell aggregates was significantly increased at higher paclitaxel concentrations (10 nM: OV-Vector 35 ± 1%/OV-KLK 23 ± 7%; 100 nM: OV-Vector 53 ± 3%/OV-KLK 38 ± 4%) compared to a lower dose (1 nM: OV-Vector 10 ± 1%/OV-KLK 9 ± 3%) and non-treated controls (0 nM: OV-Vector 6 ± 1%/OV-KLK 4 ± 2%). Interestingly, OV-KLK cell aggregates showed significantly less cell death at higher paclitaxel concentrations (10, 100 nM) than OV-Vector cells, indicating an increased cell survival (Figure 2(B), bottom panel). Over the monitored time frame of 96 h no release of trapped cells and uniform aggregation of the trapped cells ± paclitaxel treatment were evident as indicated by time-lapse microscopy (Supplementary file). As integrins are associated with cell survival and chemoresistance [30,31,47], we analyzed the expression levels of *β1 integrin* (*ITGB1*) and *focal adhesion kinase* (*FAK*), an integrin-related factor, after paclitaxel administration. In both OV-Vector/OV-KLK cell aggregates, *ITGB1* and *FAK* levels were increased upon paclitaxel treatment, with a further upregulation in OV-KLK cell aggregates (Figure 2(C)). These results suggest that hydrogel microwell arrays increase cell aggregation and viability of KLK-expressing cells upon paclitaxel treatment.

These findings are in line with our previously reported data, showing that KLK4 and KLK7 promote paclitaxel-induced resistance of ovarian cancer cell aggregates that were formed in a tumor fluid (ascites) mimicking microenvironment [25,26]. It was shown that multi-cellular aggregates, harboring a 3D architecture, are more resistant compared to flat cell cultures [48], and compact aggregates are less responsive to different therapeutic regimes, such as chemotherapies, than scattered aggregates [49]. We have also reported that combined expression of KLK4, KLK5, KLK6, and KLK7 in ovarian cancer cells (OV-KLK) mediates resistance to paclitaxel at higher doses (10, 100 nM) compared to control cells (OV-Vector) when grown as flat cell cultures [28]. When the same cells were grown as aggregates in this study, we observed a similar cell survival effect upon KLK expression and paclitaxel treatment. Interestingly, the expression of β1 integrin was decreased upon KLK expression [28], but upon paclitaxel treatment increased in both KLK-expressing and KLK-deficient aggregates, suggesting a critical function of this integrin in paclitaxel-related resistance, only partially induced by these four KLKs.

Integrins and integrin-related factors are required for the responsiveness to anti-cancer drugs that bind to microtubules [50]. Although integrins lack kinase activity, by clustering they recruit and activate kinases, such as FAK. FAK is overexpressed in most ovarian cancers, associated with poor clinical outcome and plays a role in regulating invasion and metastasis [51,52]. Paclitaxel treatment stabilizes microtubule dynamics, thereby inhibiting mitosis [40], and FAK is required for integrin-dependent microtubules stabilization and paclitaxel responsiveness [53]. It was shown that FAK regulates the efficacy of taxane-based drugs in both treatment-sensitive and treatment-resistant cells [54]. We detected increased mRNA levels of FAK in aggregates after paclitaxel treatment, further indicating that FAK is an important cell survival factor in ovarian cancer cells. These findings imply the potential of combinatorial therapeutic approaches including the inhibition of KLKs, integrin and integrin-related factors with cytotoxic drugs for the treatment of ovarian cancer patients, especially those with high KLK levels in their tumors.

### 3.3. Paclitaxel Treatment Alters Integrin Expression of Tailor-Made KLK-Expressing Cell Aggregates

**Figure 3:**
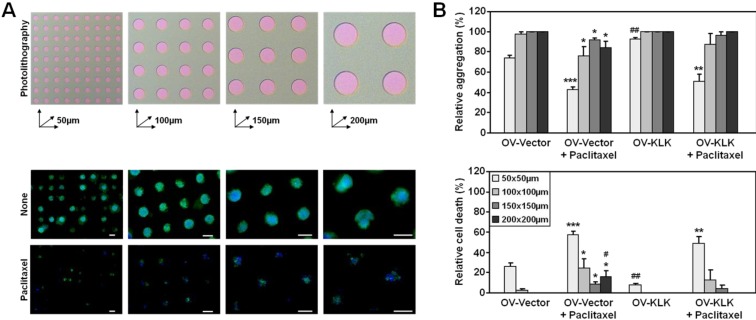
Effect of microwell size on cell aggregation and survival. (**A**) Schematic illustration of varying microwell sizes (50 × 50, 100 × 100, 150 × 150, 200 × 200 µm) generated using photolithography (top panel). Fluorescent staining of cell aggregates grown over 96 h within microwells of varying size ± paclitaxel (100 nM); F-actin filaments stained green with Alexa488-conjugated phalloidin; nuclei stained blue with DAPI (bottom panel; scale bars corresponding to respective microwell size). (**B**) Analyses of aggregation and death of both OV-Vector/OV-KLK cells depending on microwell size relative to total aggregate numbers. While in the larger microwells (100–200 × 100–200 µm) complete cell aggregation (98–100%) and no cell death (0–2%) was detected, the smallest microwells (50 × 50 µm) caused incomplete cell aggregration (73–92%) and cell death (8–27%). Upon paclitaxel treatment both OV-Vector/OV-KLK cells aggregated less (42–96%) in medium sized (100–150 × 100–150 µm) and smallest (50 × 50 µm) microwells and revealed higher cell death (4–58%) rates (n = 3; SEM; * *P* < 0.05; ** *P* < 0.01; *** *P* < 0.001). OV-KLK cells showed higher aggregation and less cell death rates in the smallest microwells (50 × 50 µm) without paclitaxel treatment and less evidence of cell death upon paclitaxel in the largest microwells (200 × 200 µm) compared to OV-Vector cells (n = 3; SEM; **^#^** *P* < 0.05; **^##^** *P* < 0.01).

Ovarian cancer cell aggregates derived from the tumor fluid (ascites) of patients with late-stage stage disease range in number (from two to more than 20) and size (from 30–200 µm, even up to 750 µm in diameter) and contain up to 100 cells, suggesting a high patient to patient variability [35,55,56,57]. This high variability in aggregate size is also reflected in *in vitro* aggregate cultures applying the liquid overlay technique [35,48,57,58] or hanging droplet method [46,59] using different ovarian cancer cell lines [34]. In order to control the cellular microenvironment of hydrogel microwell arrays, photolithography was used to fabricate microwells of varying sizes (50 × 50, 100 × 100, 150 × 150, 200 × 200 µm) to generate aggregates of different sizes (Figure 3(A), top panel). Cell aggregation was confirmed by immunostaining of F-actin filaments and nuclei (Figure 3(A), bottom panel).

**Figure 4:**
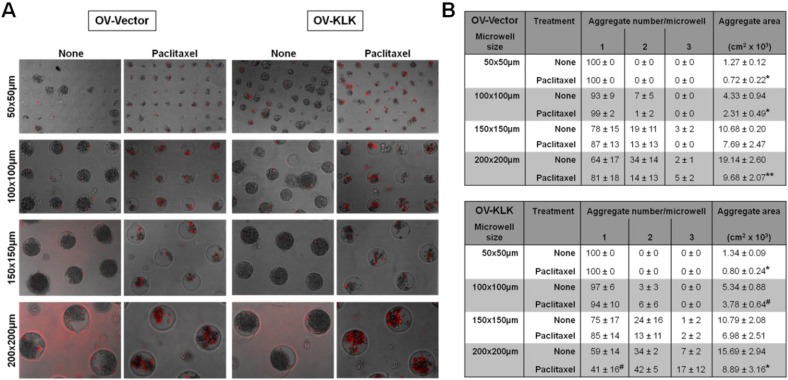
Cell aggregation as a function of microwell size, KLK expression and paclitaxel treatment. (**A**) Bright field microscopy depicted both OV-Vector/OV-KLK cell aggregates at the end time point of time-lapse microscopy carried out over 96 h within microwells of varying sizes (50 × 50, 100 × 100, 150 × 150, 200 × 200 µm) ± paclitaxel treatment (100 nM); dead cells stained red with PI. The tailor-made microwell size corresponds to respective aggregate size. (**B**) Both OV-Vector/OV-KLK cells formed one aggregate (1.27–1.34 × 10^3^ cm^2^)/microwell in the smallest (50 × 50 µm) microwells, whereas in the next larger (100 × 100 µm) microwells two aggregates (4.33–5.34 × 10^3^ cm^2^)/microwell were formed. The medium sized (150 × 150 µm) and largest (200 × 200 µm) microwells caused three aggregates (10.68–19.14 × 10^3^ cm^2^)/well. Administration of paclitaxel reduced aggregate area compared to non-treated conditions (n = 3; SEM; * *P* < 0.05; ** *P* < 0.01). Upon paclitaxel treatment both OV-Vector/OV-KLK cells formed one aggregate (0.72–0.80 × 10^3^ cm^2^)/microwell in the smallest (50 × 50 µm) microwells, while in the next larger (100 × 100 µm) microwells two aggregates (2.31–3.78 × 10^3^ cm^2^)/microwell were detected. The medium sized (150 × 150 µm) and largest (200 × 200 µm) microwells caused three aggregates (6.98–9.68 ×10^3^ cm^2^)/microwell. OV-KLK cells formed larger aggregates in the second smallest (100 × 100 µm) microwells after paclitaxel treatment compared to OV-Vector cells (n = 3; SEM; **^# ^***P* < 0.05).

As paclitaxel is subject to multi-cellular-mediated resistance for ovarian cancer cells [48], we treated tailor-made aggregates with paclitaxel (100 nM), and analyzed the aggregation and death of both OV-Vector/OV-KLK cells relative to the total numbers (Figure 3(B)). While in the larger microwells (100–200 × 100–200 µm) complete cell aggregation (98–100%) and no cell death (0–2%) was detected, the smallest microwells (50 × 50 µm) caused incomplete cell aggregration (73–92%) and cell death (8–27%). Upon paclitaxel treatment both OV-Vector/OV-KLK cells aggregated less (42–96%) in the medium sized (100–150 × 100–150 µm) and smallest (50 × 50 µm) microwells and showed higher cell death (4–58%) rates (Figure 3(B)). Then, we analyzed the aggregate number and area in each microwell size performing time-lapse microscopy (Figure 4(A)). Both OV-Vector/OV-KLK cells formed one aggregate (1.27–1.34 × 10^3^ cm^2^)/well in the smallest (50 × 50 µm) microwells, whereas in the next larger (100 × 100 µm) microwells, two aggregates (4.33–5.34 × 10^3^ cm^2^)/well were formed. In the medium sized (150 × 150 µm) and largest (200 × 200 µm) microwells, three aggregates (10.68–19.14 × 10^3^ cm^2^)/well were formed. Upon paclitaxel treatment both OV-Vector/OV-KLK cells formed one aggregate (0.72–0.80 × 10^3^ cm^2^)/well in the smallest (50 × 50 µm) microwells, while in the next larger (100 × 100 µm) microwells, two aggregates (2.31–3.78 × 10^3^ cm^2^)/well were detected. In the medium sized (150 × 150 µm) and largest (200 × 200 µm) microwells, three aggregates (6.98–9.68 × 10^3^ cm^2^)/well were formed. OV-KLK cells formed larger aggregates in the second smallest (100 × 100 µm) microwells after paclitaxel treatment compared to OV-Vector cells (Figure 4(B)). These results suggest that OV-KLK cells had a higher ability to aggregate and survive with and without paclitaxel in all microwell sizes compared to OV-Vector cells. The administration of paclitaxel reduced aggregate area but not numbers compared to non-treated conditions.

Within these bioengineered microwells, the formation of cell aggregates was achieved in sizes ranging from 50–200 µm. Similar aggregate sizes are described in experimental and clinical samples [35,55,56,57] showing a high cell viability in combination with KLK expression, and the results presented in this study are in line with our former reports [25,26]. Paclitaxel treatment revealed that the aggregate area but not aggregate number was reduced, further corroborating the existence of survival-promoting factors, such as integrins, and multi-cellular-mediated drug resistance mechanisms in ovarian cancer cells [48]. A similar bioengineered approach to the one described here has been used to control the size and shape of embryonic bodies employing microwells of varying diameters ranging from 40–150 µm and heights of 20–35 µm and has proven its potential to investigate differentiation of embryonic stem cells [60]. These findings indicate that hydrogel microwell arrays can be used to control cell aggregation, aggregate size and viability, to study factors involved in the responsiveness of different sized aggregates to anti-cancer drugs and the contribution of KLKs.

**Figure 5:**
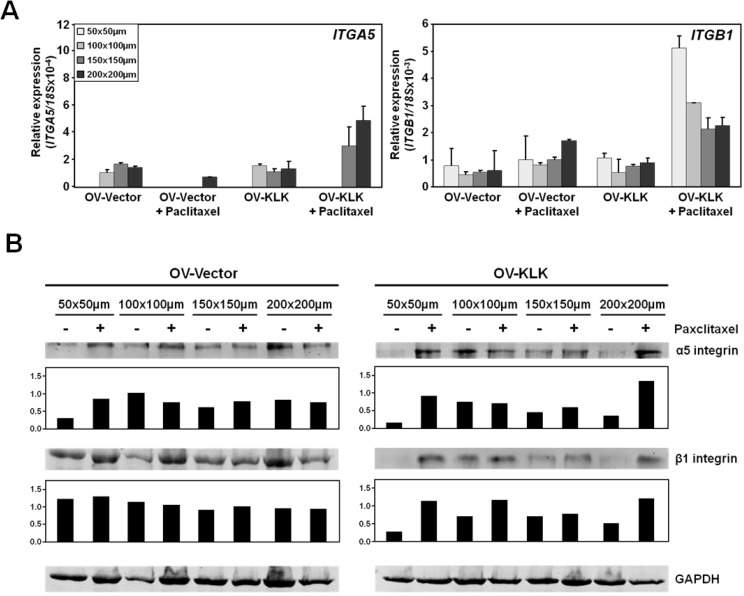
Altered expression levels as a function of microwell size, KLK expression and paclitaxel treatment. (**A**) Levels of *ITGA5* were a function of microwell size and KLK expression after paclitaxel treatment (100 nM), with highest expression in aggregates grown in the largest (150–200 × 150–200 µm) microwells. No difference in both OV-Vector/OV-KLK cell aggregates without treatment was detected. Levels of *ITGB1* were enhanced after paclitaxel administration in both OV-Vector/OV-KLK cell aggregates, with highest expression on OV-KLK cell aggregates in the smallest (50–100 × 50–100 µm) microwells. No difference in both OV-Vector/OV-KLK cell aggregates without treatment was detected. (**B**) Western blot and densitometrical analyses demonstrated that α5 and β1 integrin expression was enhanced after paclitaxel treatment in OV-KLK cell aggregates compared to OV-Vector cells which only showed an increase of α5 integrin in the smallest (50 × 50 µm) and medium sized (150 × 150 µm) microwells.

Integrins are integral in mediating cell survival and chemoresistance, in particular α5/β1 integrins [30,31,47]. Hence, we sought to determine α5/β1 integrin mRNA and protein levels in aggregates of varying size upon paclitaxel treatment (100 nM). While no difference in both OV-Vector/OV-KLK cell aggregates without treatment was found, after paclitaxel treatment *ITGA5* was increased in aggregates grown in the largest (150–200 × 150–200 µm) microwells, and *ITGB1* was enhanced in aggregates, with highest expression levels in OV-KLK cell aggregates grown in the smallest (50–100 × 50–100 µm) microwells (Figure 5(A)). Western blot and densitometrical analyses showed that α5 and β1 integrins were enhanced after paclitaxel treatment in OV-KLK cell aggregates compared to OV-Vector cells, which only had increased α5 integrin in the smallest (50 × 50 µm) and medium sized (150 × 150 µm) microwells (Figure 5(B)). Interestingly, the biggest (200 × 200 µm) microwells resulted in multiple smaller aggregates per microwell (34%), which have the same integrin expression pattern as the aggregates formed in the smallest (50 × 50 µm) microwells. These results suggest that integrin expression is upregulated upon paclitaxel treatment depending on the aggregate size and partially on KLK expression, especially in smaller (50 µm) and larger (150–200 µm) aggregates.

**Figure S1:**
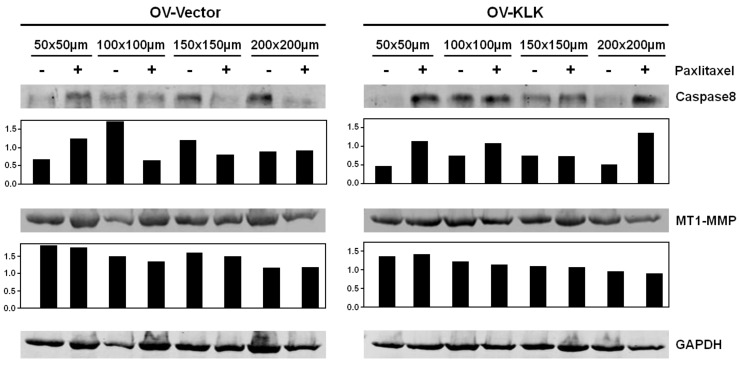
Altered caspase8 expression levels as a function of microwell size, KLK expression and paclitaxel treatment. Caspase8, indicative of apoptosis, was expressed in aggregates grown in the four different microwell sizes (50 × 50, 100 × 100, 150 × 150, 200 × 200 µm) without paclitaxel treatment and upregulated in OV-KLK cell aggregates upon paclitaxel administration. OV-Vector cell aggregates showed a downregulation of capsase8 in medium sized (100–150 × 100–150 µm) microwells upon paclitaxel treatment. Caspase8 levels did not change in both OV-Vector/OV-KLK cell aggregates in the second largest (150 × 150 µm) and largest (200 × 200 µm) microwells respectively upon paclitaxel treatment. Altered MT1-MMP expression levels as a function of microwell size. MT1-MMPwas expressed in all conditions (50 × 50, 100 × 100, 150 × 150, 200 × 200 µm) independent of KLK expression and paclitaxel treatment.

Caspases play an important role in apoptosis induced by anti-cancer drugs [61]. In both OV-Vector/OV-KLK cell aggregates, caspase8 expression followed β1 integrin levels in the smallest (50 × 50 µm) and largest (200 × 200 µm) microwells. OV-Vector cell aggregates showed a downregulation of capsase8 in medium sized (100–150 × 100–150 µm) microwells upon paclitaxel treatment (Figure S1). These results imply an involvement of integrins in paclitaxel-induced apoptosis. However, our findings suggest a bi-functional effect of drug treatment: (i) upregulation of integrins to promote cell aggregate survival, and (ii) upregulation of caspase-8 to mediate cell death, further underlining the fine-tuned balance between drug sensitivity and drug resistance.

It was shown that the membrane type 1 matrix metalloproteinase (MT1-MMP) regulates ovarian cancer cell aggregation and disaggregation, and its expression level is increased in aggregates relative to flat cell cultures [43]. Ovarian cancer cell aggregates grown within microwells of varying sizes showed MT1-MMP expression in all aggregate sizes independent of KLK expression and paclitaxel treatment (Figure S1). MT1-MMP can be regulated by integrin clustering which was shown to be stimulated by a 3D collagen type I microenvironment [62]. In addition to MT1-MMP activity [43], other factors, such as contractile forces [59], promote cell aggregation. The simultaneous presence of MT1-MMP and integrins in aggregates grown within hydrogel microwell arrays further indicates their interactive relationship within this microarray platform.

### 3.4. Blocking of Integrin Function Does Not Affect Cell Aggregation

**Figure 6:**
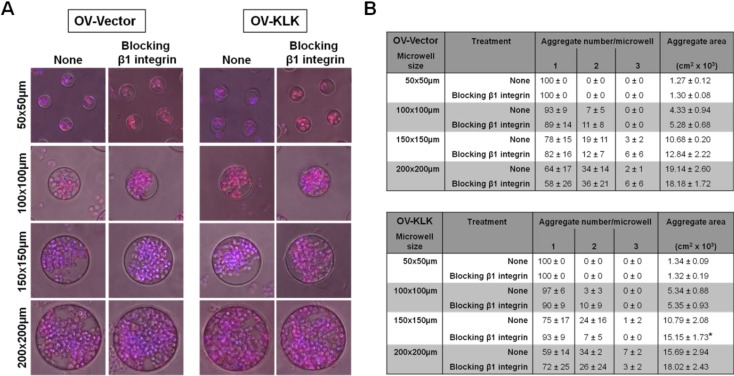
Cell aggregation in response to functional blocking of integrins. (**A**) Fluorescent staining of cell aggregates grown within microwells of varying sizes (50 × 50, 100 × 100, 150 × 150, 200 × 200 µm) over 96 h at the end time point of time-lapse microscopy ± inhibition of β1 integrin using a functional blocking antibody (10 µg/mL); F-actin filaments stained red with rhodamine 415-conjugated phalloidin; nuclei stained blue with DAPI. (**B**) Analyses of both OV-Vector/OV-KLK cell aggregate number and area revealed that with increasing microwell size (150–200 × 150–200 µm) more (up to three aggregates/microwell) and larger (12.84–18.18 × 10^3^ cm^2^) aggregates were formed upon β1 integrin inhibition compared to decreasing microwell size (50–100 × 50–100 µm; 1.30–5.35 × 10^3^ cm^2^), with only one to two aggregates/microwell. In the medium sized microwells (150 × 150 µm), OV-KLK cells formed significantly larger aggregates after β1 integrin inhibition compared to non-treated conditions (n = 3; SEM; * *P* < 0.05).

It was shown that β1 integrin regulates the formation of ovarian cancer cell aggregates that were generated using the liquid overlay technique [35,55,56]. Hence, we sought to test whether the formation OV-Vector/OV-KLK cell aggregates produced in hydrogel microwell arrays is dependent on β1 integrin by using a functionally blocking antibody (10 µg/mL). Surprisingly, both OV-Vector/OV-KLK cell aggregate number and area was enhanced with increasing microwell size (150–200 × 150–200 µm) upon integrin inhibition, with more (up to three aggregates/well) and larger (12.84–18.18 × 10^3^ cm^2^) aggregates being formed compared to non-treated conditions. With decreasing microwell size (50–100 × 50–100 µm), only one to two aggregates/well and smaller aggregates (1.30–5.35 × 10^3^ cm^2^) were formed. In the medium sized microwells (150 × 150 µm), OV-KLK cells formed significantly larger aggregates after integrin inhibition compared to non-treated conditions (Figure 6(A,B)).

Different to the study by Casey *et al*. [35], which reported the inhibition of aggregation using the same blocking β1 integrin antibody after 8 h and 24 h in serum-free media, is that we documented the integrin inhibition over 96 h in serum-containing media. Casey *et al.* [35] showed that after 8 h aggregate formation was inhibited by the blocking β1 integrin antibody, resulting in none or small aggregates. At 24 h, β1 integrin inhibition continued to partially block aggregate formation, resulting in medium to large aggregates. The incomplete inhibition of the β1 integrin at the 24 h time point suggests that if this integrin is inactivated, ovarian cancer cells might possess a compensatory mechanism to facilitate aggregation. However, the antibody might have been internalized over 24 h and 96 h, eventually enabling ovarian cancer cells to aggregate. Moreover, the presence of the serum-containing media allows the continuous proliferation of cells over a longer period of time. It was suggested that β1 integrin mediates the initial formation of cell aggregates and that multiple integrin-ECM interactions, such as αv integrin/vitronectin [57], are involved in this process.

Contrary to Casey *et al.* [35] we hypothesized that aggregation time and technique are important parameters. Casey *et al*. [35] demonstrated that NIH:OVCAR5 cells formed stable aggregates within 48 h using the liquid overlay method, whereas the OV-MZ-6 cells used in our study formed compact aggregates for up the 120 h within hydrogel microwell arrays. In our previously published work, we demonstrated that OV-MZ-6 cell spheroids proliferated for up to 28 days [8], underlining the robustness of this cell line when combined with a biomimetic hydrogel in a high-throughput system. Although the capacity to form compact aggregates differs between ovarian cancer cell lines [8,35,57], the aggregates formed in bioengineered microenvironment emerge to be similar to those present in the tumor fluid (ascites) of patients.

## 4. Conclusions

When entering the third dimension, investigators need to consider the design of microenvironments for supporting the cell architecture and the capability to conduct such a system in high-throughput. We provide evidence that hydrogel microwell arrays can be engineered to replicate intricate biological functions the tumor microenvironment by allowing aggregation of ovarian cancer cells, and thus, are well suited to decipher the function of cancer-associated proteases and integrins in disease progression and therapy-resistance. Tailor-made hydrogel microwells increase cell aggregation and insensitivity to paclitaxel treatment, in particular in KLK-expressing cancer cells, and thus, representing events seen in patients with metastatic outgrowth. KLK expression in cancer cell aggregates was accompanied with altered integrin levels and integrin-related factors upon paclitaxel treatment. However, blocking of integrin function did not affect cancer cell aggregation, suggesting that the involvement of other cell surface molecules and/or receptors play an important role. In conclusion, the technology platform presented in this study has the potential to provide an alternative screening tool for the efficacy of novel therapeutics specifically targeting multi-cellular aggregates for intra-abdominal intervention of late-stage disease.

## Supplementary Materials

Supplementary File 1

## Appendix

**Supplementary files.** Time-lapse microscopy of aggregation dependent on paclitaxel treatment. Representative time-lapse experiments (avi-files) of aggregates grown under non-treated (2009.07.30_Overlay_s43_KLK) and treated (2009.07.30_Overlay_s53_KLK+Taxol.avi) conditions are shown using a widefield microscope over 96 h, with images taken every 6 h using a 10× air objective.
